# Discovering environmental management opportunities for infectious disease control

**DOI:** 10.1038/s41598-021-85250-1

**Published:** 2021-03-19

**Authors:** Ludovica Beltrame, Hannah Rose Vineer, Josephine G. Walker, Eric R. Morgan, Peter Vickerman, Thorsten Wagener

**Affiliations:** 1 Department of Civil Engineering, University of Bristol, Bristol, UK; 2 Department of Agricultural and Environmental Sciences, University of Milan, Milan, Italy; 3 Department of Infection Biology and Microbiomes, Institute of Infection, Veterinary and Ecological Sciences, University of Liverpool, Liverpool, UK; 4 Bristol Medical School, University of Bristol, Bristol, UK; 5 School of Biological Sciences, Queen’s University Belfast, Belfast, UK; 6 Cabot Institute for the Environment, University of Bristol, Bristol, UK; 7 Institute for Environmental Science and Geography, University of Potsdam, Potsdam, Germany

**Keywords:** Ecology, Environmental sciences, Hydrology, Diseases, Infectious diseases, Parasitic infection

## Abstract

Climate change and emerging drug resistance make the control of many infectious diseases increasingly challenging and diminish the exclusive reliance on drug treatment as sole solution to the problem. As disease transmission often depends on environmental conditions that can be modified, such modifications may become crucial to risk reduction if we can assess their potential benefit at policy-relevant scales. However, so far, the value of environmental management for this purpose has received little attention. Here, using the parasitic disease of fasciolosis in livestock in the UK as a case study, we demonstrate how mechanistic hydro-epidemiological modelling can be applied to understand disease risk drivers and the efficacy of environmental management across a large heterogeneous domain. Our results show how weather and other environmental characteristics interact to define disease transmission potential and reveal that environmental interventions such as risk avoidance management strategies can provide a valuable alternative or complement to current treatment-based control practice.

## Introduction

Many infectious diseases have strong environmental components to their transmission. The role of the environment on infectious disease dynamics varies depending on their pathways. On the one hand, transmission is mainly governed by direct host-to-host contact for disease agents that cannot survive long in the landscape (e.g. influenza). On the other hand, environmental conditions become increasingly important the longer pathogens are able to survive outside of their final host (e.g. waterborne diseases like cholera, and infections transmitted through vectors or intermediate hosts that live and develop in the environment such as malaria, schistosomiasis and fasciolosis)^1^. Crucially, many transmission routes through water, food, soil and vectors or intermediate hosts, are not only associated with meteorological drivers like air temperature and rainfall, but are also mediated by environmental and landscape characteristics, such as hydrologic transport for cholera^2^, the persistence of water pools for malaria^3^, the hydrological regime for schistosomiasis^4^ and soil moisture for fasciolosis^5^. These characteristics may be distributed unevenly in space, change rapidly over time, act at different scales, and directly and/or indirectly affect multiple disease components, often resulting in complex disease dynamics^1,6–8^.

Controlling environmentally transmitted diseases is becoming more and more challenging. For many of these infections, no commercial vaccines are yet available for prevention, and control relies entirely on drug administration. However, as long as environmental conditions remain suitable for transmission, reinfection may occur rapidly after treatment^9^. Moreover, the widespread emergence of drug resistance—due to overreliance on a single medicine—is threatening the efficacy and sustainability of current treatment-based control strategies for an increasing range of infections such as schistosomiasis, fasciolosis and other Neglected Tropical Diseases, globally^9–13^. Finally, for many diseases, this problem is aggravated by the frequent misuse and overuse of drugs linked to altered epidemiological patterns (such as the emergence of infections at new times/places) caused, at least partly, by climate and land use changes^14–17^. In fact, the response to an increased disease challenge is often an increased use of treatment, which accelerates development of drug resistance^18^.

As climate change accelerates and disease agents become ever more resistant to drugs, the devising of more holistic strategies—rather than exclusively relying on treatment—is becoming a key concern. The role that environmental conditions play in driving disease transmission may offer opportunities to use environmental interventions as complementary—or even as alternative—strategies to drug administration to reduce disease burdens^9,19^. To be able to explore the potential of environmental management for risk reduction, models based on a better mechanistic understanding of the link between disease transmission and underlying drivers are needed^20–25^. In fact, testing the impact of new potential environmental control strategies on large-scale current and future disease transmission cannot be performed in the field, but requires mechanistic models for developing what-if analyses^26,27^. Crucially, such models need to include representation of on-the-ground environmental characteristics (beyond meteorological variables alone), which are those that often directly control epidemiological processes (at least for the transmission routes through water/food/soil/vectors/hosts mentioned above), as well as those that decision-makers might be able to manipulate locally to contribute to sustainable and effective control^1,19,28^.

However, while the role of on-the-ground environmental processes in mediating disease risk responses to weather factors is increasingly acknowledged (e.g. ^2–4,25,28,29^, the majority of current studies either only consider them empirically (e.g.^30–32^), or assume meteorological drivers of disease risk only (e.g.^33–35^), usually focusing on temperature and rainfall characteristics alone^6,15,23,36^. Arguably, partly as a consequence of this, despite showing promising results in the fight against diseases like schistosomiasis^28,37^, strategies targeting the environmental stage of the pathogen, to complement medical approaches, are still poorly developed^9^.

Therefore, in this study, focusing on fasciolosis in the UK, we explore opportunities for environmental management as a control strategy through mechanistic modelling, while considering the diversity of disease drivers across this heterogeneous domain. While rapidly emerging in humans in other parts of the world, fasciolosis in the UK mainly affects livestock, where it currently costs the agriculture sector approximately £300 M per year due to lost production^38^. Moreover, considerable changes in its seasonality and spread have been observed in recent decades and attributed to changing climatic patterns^14,39–41^. In fact, a significant proportion of the life cycle of liver fluke, the parasite responsible for the disease, takes place in the environment and is strongly affected by soil moisture (which varies with factors like topography and rainfall) and air temperature. Importantly, these factors determine habitat suitability for the liver fluke’s amphibious intermediate snail hosts and thus control disease risk, with saturated soil and moderate temperatures favoring transmission (^5,42^; SI Appendix). As resistance towards available drugs against fasciolosis is increasingly reported, the possibility to avoid infection by disrupting the parasite life cycle and reducing environmental suitability for disease transmission has been often advocated^11,12^. Though the practical difficulties of evaluating the effectiveness of such interventions in the field, while controlling for cofounding factors, and previous lack of environment-based mechanistic liver fluke models for what-if analyses at relevant scales, have not allowed stakeholders to fully explore the potential of environmental management for risk reduction. Here, we simulate the risk of liver fluke infection for the recent period 2006–2015 over 935 UK catchments using the newly developed and validated mechanistic Hydro-Epidemiological model for Liver Fluke (HELF,^24^). We assess disease risk sensitivity to underlying environmental drivers by estimating the contribution of meteorological and topographic characteristics, and their two-way interactions, to seasonal disease risk across 9 administrative regions, using ANalysis Of VAriance (ANOVA). Finally, we investigate the potential of environmental strategies by analyzing where they can provide benefits in terms of risk reduction and how they compare with current treatment-based control. Specifically, (i) we evaluate the effect of using the most efficient drugs currently available (assuming no resistance) on risk of infection^43,44^, and (ii) we assess the effectiveness of two environmental interventions that have been studied empirically, but whose potential impact on disease transmission has not yet been quantified across larger regions: fencing off high-risk areas to prevent livestock from grazing at high-risk times, and soil drainage^11,18,45–47^.

## Results

### Simulated UK-wide disease risk

**Figure 1:**
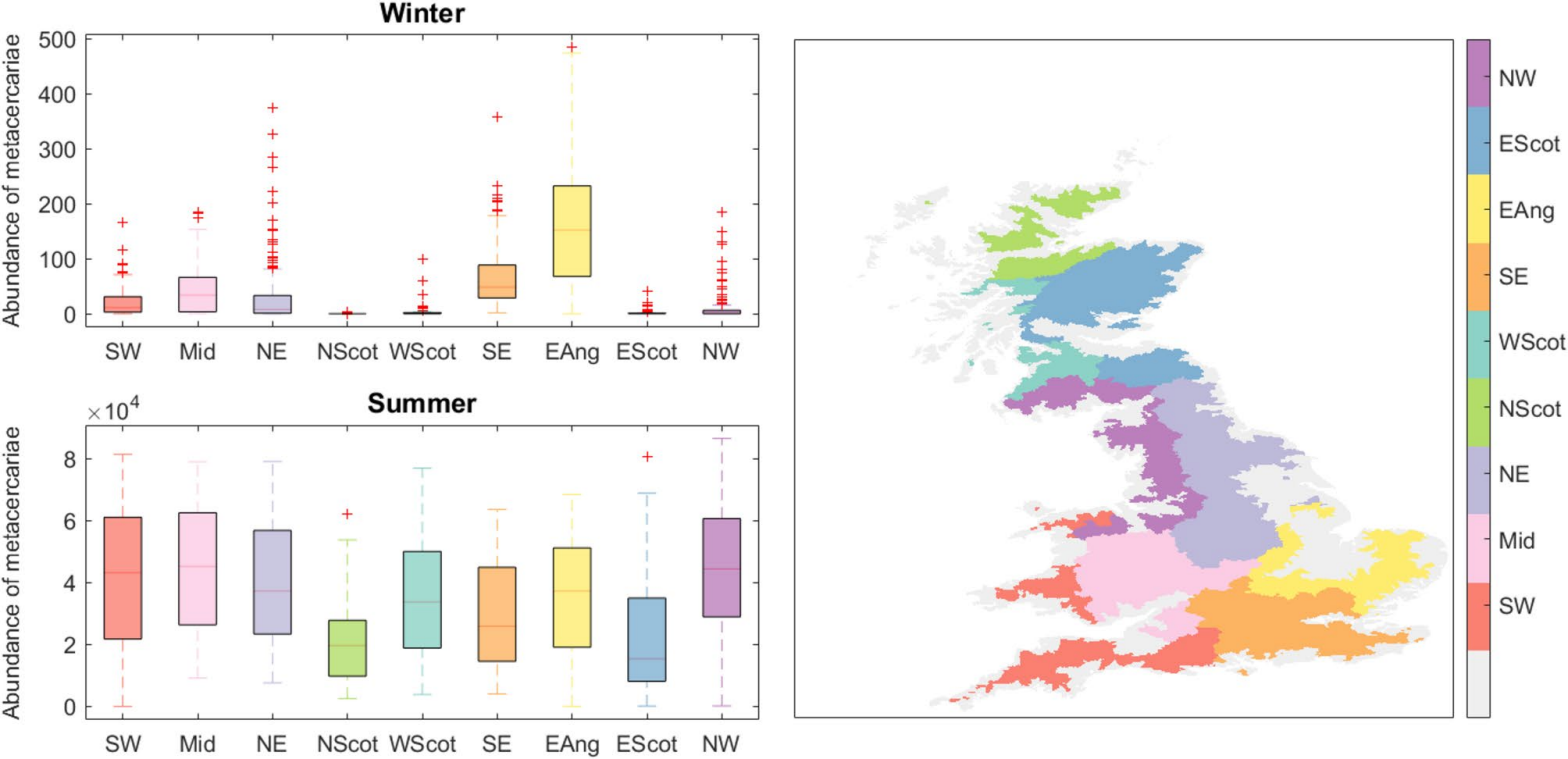
Seasonal abundance of metacercariae, i.e. disease risk simulated using HELF, on average over 2007–2015, across 9 UK regions. Boxplots represent variability in disease risk between catchments within regions. In the map (created using Matlab R2019a), ungauged catchments -i.e. with no hydrological data over the simulation period- are masked in grey. *SW* South West of England and West Wales, *Mid* rest of Wales and Midlands, *NE* North East of England, *NScot* North of Scotland, *WScot* West of Scotland, *SE* South East of England, *EAng* East Anglia, *EScot* East of Scotland, *NW* North West of England.

Disease risk is quantified in terms of the abundance of metacercariae on pasture, i.e. the parasite life-cycle stage that is infective to the livestock hosts. Risk values simulated with HELF across Great Britain reveal differences between winter and summer (Fig. 1). While in winter the only regions where weather conditions allow the presence of infective metacercariae on pasture are those in the south of the country, where temperatures are milder, risk values are significantly higher over all regions in summer. Specifically, summer median risk levels are highest on the west coast of England and Wales (where rainfall is abundant even during the warmer months), lower in the South East of the country, which in summer can become too warm and dry for parasite survival and development, and even lower in Scotland, where, even during milder summer months, temperatures can still be relatively unfavorable. These results suggest that weather factors are important controls on risk of infection and are in agreement with current understanding and data. For example, one of the largest UK studies on liver fluke prevalence found the highest infection levels in wetter western areas of the country, which historically have been providing ideal weather conditions for disease transmission^41^. It is also interesting to note that, within regions, simulated summer risk values are highly variable, especially in South West of England and West Wales (SW) and North West England (NW). In fact, even if, in summer, risk values are generally high, we see that there still can be areas associated with lower abundance of metacercariae. This can be due to different dynamic weather effects on development within the parasite life cycle, but also to landscape heterogeneities (e.g. areas at the bottom of a valley will be more prone to saturation and therefore at higher risk compared to areas with similar weather patterns but located on a steep hill), and interactions between the two. This also is in agreement with existing datasets, which show significant differences in disease prevalence between neighboring areas within homogeneous climatic regions, already suggesting that other factors may affect the prevalence of liver fluke in addition to meteorological conditions^41^.

### Disease risk sensitivity to environmental drivers

**Figure 2:**
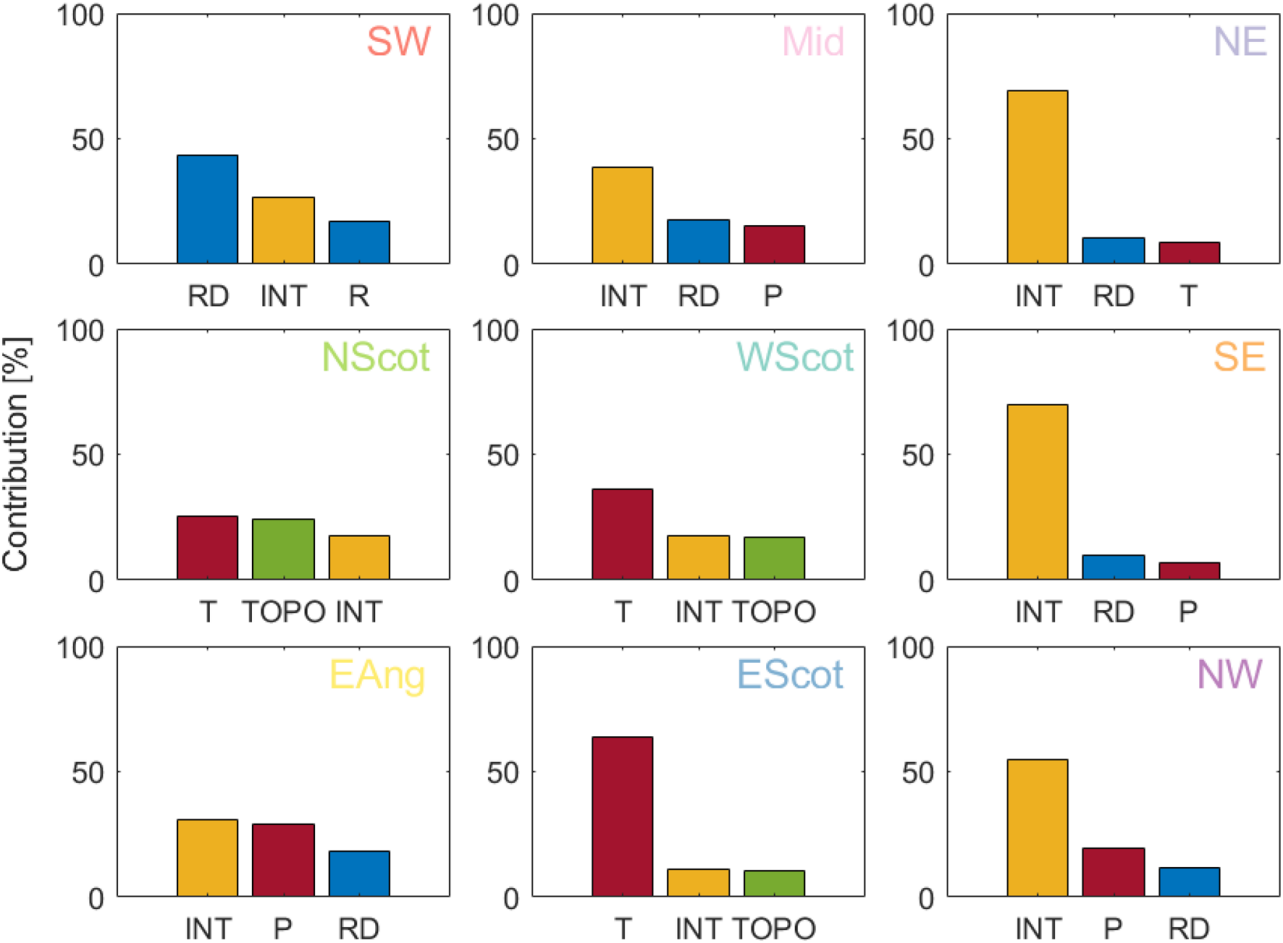
Average percent contribution of the top three environmental drivers to summer disease risk variability simulated using HELF, per region. Rainfall-related characteristics are in blue (RD = number of rainy days and R = rainfall); temperature-related variables are in red (P = potential evapotranspiration and T = temperature); landscape factors are in green (TOPO = topography); and two-way interactions between factors (here combined into a single term INT) are in yellow. Regions are defined in Fig. 1.

Results of ANOVA at the regional level identify the main controlling factors of disease risk across areas and indicate reasons for the large variability we see in summer. Figure 2, which presents the identified top three drivers of summer disease risk per region, shows that temperature (T, in red) is more important in northern areas and is the main control on disease risk in Scotland. Whereas, further south, variability in rainfall-related characteristics (in blue) explains more of the risk differences between catchments. Importantly, two-way interactions between analyzed factors (which in Fig. 2 are combined into a single term, INT, in yellow) play a significant role on disease transmission across all areas. In fact, INT appears as dominant control in 5 out of 9 regions, suggesting that, without considering interactions, the importance of individual factors may be overestimated. While in flat low-lying areas in the south east of the country (South-East of England and East Anglia; Figure S1), it is the interaction between weather factors themselves that explains most of the variability in risk, along the west coast of England and Wales disease risk shows higher sensitivity to interactions between meteorological characteristics and topography (Table S1). Potentially less limiting weather conditions in these areas compared to the south east (i.e. less extreme warm temperatures and more abundant rainfall), coupled with a higher topographic variability (i.e. presence of both flat and hilly terrain), result in a stronger role of topography in modifying meteorological impacts, with flatter areas in valleys saturating more easily, favouring intermediate host habitats and thus disease transmission. Notably, in the Scottish regions, among the coldest and wettest of the country, while disease risk is mainly limited by temperature, topography (TOPO, in green) also individually emerges as an important driver, with steep vs. flat terrain creating differences in disease risk even in areas close to each other, highlighting potential opportunities for disease control through environmental management.

### Efficacy of current treatment-based control

**Figure 3:**
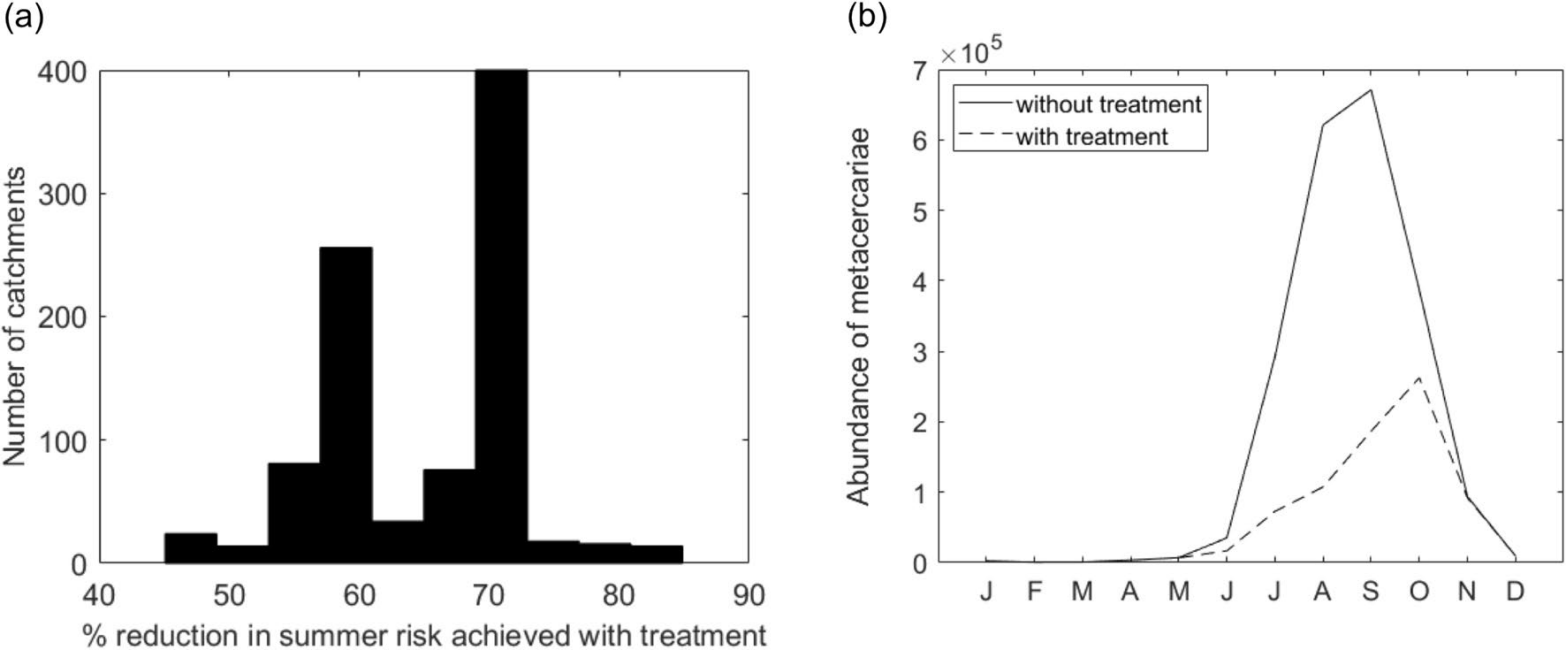
Effect of treating livestock twice per year (in January and April) using the current most efficient antiparasitic drug (90%) and assuming no resistance, on: (**a**) summer disease risk across all 935 analyzed catchments; (**b**) the monthly abundance of infective metacercariae on pasture (i.e. disease risk), on average across all catchments and years.

Figure 3 shows the effect on risk of infection (simulated using HELF) of treating animals twice per year (in January and April) using the most efficient drug currently available (90% efficacy) assuming no resistance. On average, this strategy achieves a reduction in disease risk over summer of 65% (Fig. 3a). For most catchments, risk is even reduced by more than 70%. However, due to differences in weather-environmental conditions and their seasonality, treatment can have different effects on the abundance of infective metacercariae, and therefore on disease risk, across our domain (range: 45–84.9%). On the other hand, if we look at the impact of treatment on disease risk in time (Fig. 3b), we see that, in addition to risk being significantly reduced, the rise in metacercarial abundance on pasture is also delayed (by approximately one month) and, similarly, the peak of infection is shifted to later in the year (approximately from August–September to October), as a result of drug administration. Overall, these results agree with experience and suggest that treatment-based control would be effective in limiting risk across UK regions, if changing climatic and weather patterns were not increasing opportunities for transmission, and if resistance to available drugs was not developing rapidly^12,14^.

### Effectiveness of environmental interventions

**Figure 4:**
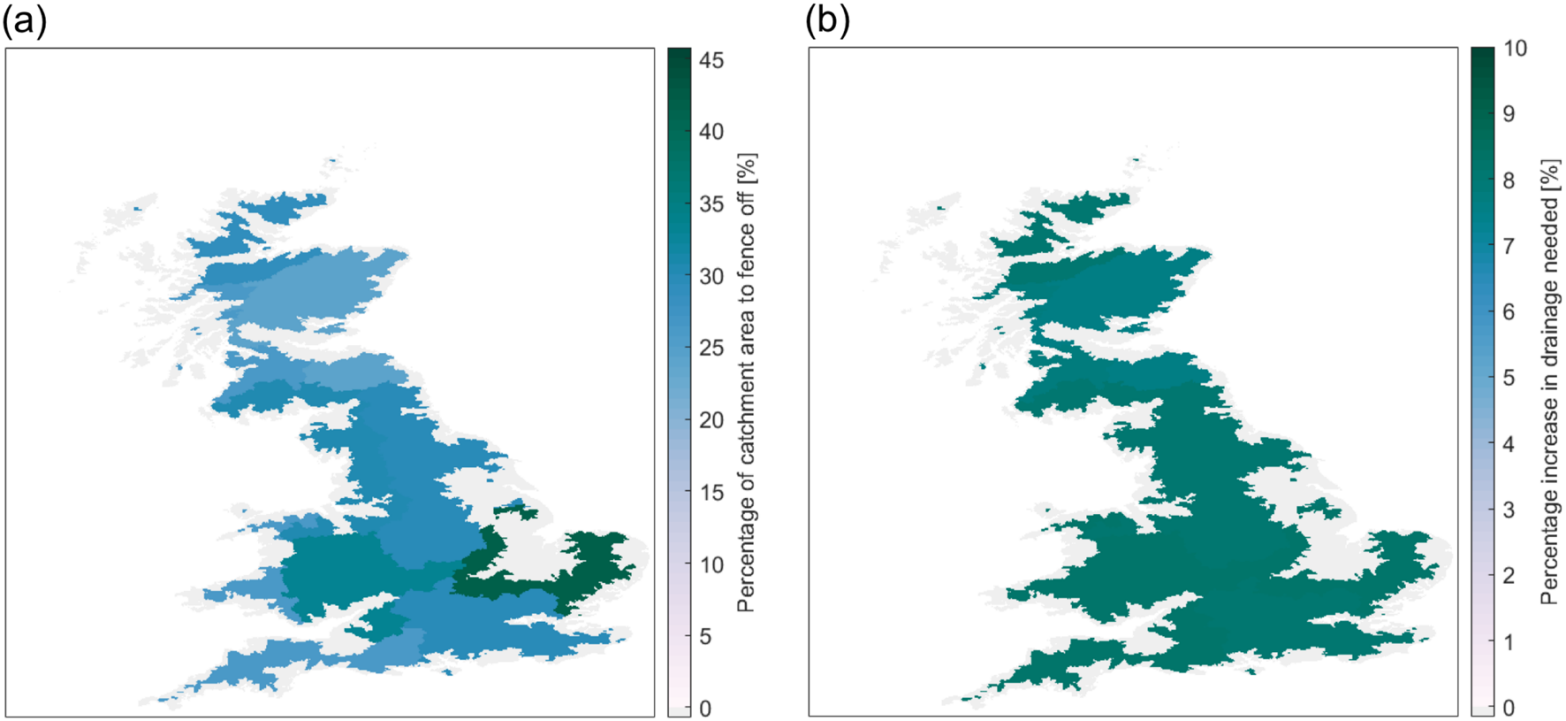
Effectiveness of environmental interventions (maps created using Matlab R2019a): (**a**) Percentage of catchment area we would have to exclude from livestock grazing to reduce summer risk of infection by at least the same percentage achieved using treatment (on average across catchments within each region and for year 2013 as an example within our simulation period—but also see Figure S2 for a comparison with a wetter year); (**b**) Percentage increase in drainage that would be needed, compared to current conditions, to match as close as possible the reduction in summer risk of infection achieved using treatment (on average across catchments within each region).

Simulating disease risk using HELF also allows us to investigate how different sensitivities to environmental drivers may translate into different opportunities for risk reduction through environmental management across areas. For example, summer risk of infection can be reduced by temporarily excluding grazing livestock from high-risk fields, though our results show that the percentage of catchment area that must be fenced off, to match the level of risk reduction achieved by drug treatment, varies from 23.8 to 42.1% across UK regions (Fig. 4a, but also see Figure S2 for a comparison between a dry and a wet year). In each catchment, this strategy consists of fencing off areas *starting from those most prone to saturation, which are those most likely to provide favorable habitats for snail intermediate hosts, and therefore most suitable for disease transmission* (i.e. flat areas at the bottom of valleys). Overall, the lowest percentages are found in Scotland (23.8%), where topographic variability is highest and thus snail habitats will be most localized, as well as where risk of infection is generally lower due to less favorable temperatures. In the comparatively warmer but still hilly areas of the South-West of England and West Wales, the percentage of land to be avoided is similar (26.3%), confirming that fencing may be particularly helpful in regions with larger topographic variations, rather than where the landscape is mostly flat^47^. In contrast, the highest fractions of land over which grazing should be avoided are located in the south east of our domain, specifically in East Anglia, where 42.1% of catchment area has to be fenced off, on average. Areas in this region are among the driest and warmest of the country, which would suggest relatively limited risk over summer and therefore limited need for fencing compared to the west coast. However, these areas are also the flattest part of Great Britain (Figure S1), which explains why fencing off large portions of land to isolate saturated fields is necessary to match the effect of treatment. In fact, whenever weather conditions allow, saturated areas and snail habitats will be widespread rather than confined to valleys at the bottom of hills or mountains as in higher relief regions. As an alternative to temporary fencing, Fig. 4b shows, for each region, the percentage increase in soil drainage that would be needed in order to match as closely as possible the reduction in summer risk of infection achieved using treatment. We find that with this other environmental intervention, *which in our study -in contrast to fencing- is implemented at the catchment level*, differences in terms of opportunities for risk reduction across UK areas are not as pronounced. In fact, while, as with fencing, more drainage would be required in the warmer south of the country rather than in Scotland (where, despite rainfall not being limiting, temperatures remain comparatively unfavorable), the increase in drainage needed to match treatment is approximately 8% compared to current conditions over all analyzed regions. Nevertheless, this result suggests that, in principle, at least where large percentages of catchment area would have to be fenced off, drainage could provide a valuable alternative to current treatment-based control.

## Discussion

This study is the first to consider environmental controls on fasciolosis, beyond just weather characteristics, across a large heterogeneous domain, and to do so in a mechanistic (rather than empirical/correlation-based) manner. Specifically, instead of only focusing on temperature and rainfall-related variables, as in previous correlation-based large-scale liver fluke modelling studies (e.g.^33–35^), we also account for soil moisture patterns, which are assumed to vary with heterogeneous topography, and directly control habitat suitability for parasite development and disease transmission^5,42^. Moreover, in contrast to existing studies that do include environmental controls and management in the investigation of risk factors at specific times and locations (e.g.^30–32^), we consider these mechanistically. This represents the first step towards assessing opportunities for environmental management as a disease control strategy across the UK and beyond. In fact, on-the-ground environmental characteristics, as opposed to climate and weather patterns, may be modifiable or “reasonably amenable to management or change given current knowledge and resources” towards reducing infection levels^19,28^. Furthermore, it is only through mechanistic modelling that management scenarios and their impacts on disease transmission can be evaluated at a large scale and over long time horizons, under current and future potential conditions^20–27^, which is urgently needed for supporting long-term planning and policy-making at the national level (e.g. see the UK National Animal Disease Information System, NADIS^48^).

Our simulations of liver fluke risk over UK catchments, and our regional analysis of disease sensitivity to environmental factors, show that, while weather remains a key driver across the country, topography emerges as an important control in specific areas (Figs. 1, 2). First, the fact that drivers other than meteorological ones come into play when moving towards more regional levels, confirms speculations in previous empirical studies on fasciolosis, as well as findings for other environment-driven diseases. Crucially, Fox et al. warn about using liver fluke risk forecasting models which only account for climatic drivers (e.g.^42^) when focusing on regional levels, as, at these scales, many non-climatic factors become relevant in driving parasite survival and transmission^34^. Similarly, Liang et al. find that, within a climatologically homogeneous region in China, land use and characteristics of the irrigation system are the main drivers of human infection with schistosomiasis^28^. Second, the fact that landscape heterogeneity may alter risk of liver fluke infection, and its sensitivity to climatic/meteorological variability, suggests that future UK disease control strategies will need to explicitly account for it. Specifically, simulations of disease risk based on models with no representation of landscape heterogeneities (such as the empirical Ollerenshaw Index (^42^, SI), the basis of current industry standard forecasts^48^) may have limited utility over regions with more pronounced topographic variability going forwards. Finally, our result that landscape heterogeneity most often exerts its control on disease risk through interaction with weather variables is consistent with current knowledge of how the parasite life cycle depends on amphibious intermediate snail hosts, which live on saturated areas, that vary with topography^5,42^. On the other hand, the fact that regional between-catchment variation in risk is explained by different environmental characteristics, and interactions between them, suggests that regionalized control policies and practices may be more effective than one-size-fits-all national strategies.

Having recognized the role of landscape heterogeneity in driving risk of infection alongside meteorological factors, our findings on the effectiveness of environmental interventions have important implications for disease control. While the current European farm subsidy structure -whereby loss of productive grazed land would be financially penalized- may provide a disincentive to its implementation, fencing off high-risk areas to avoid grazing during high-risk periods has been often called for as an aid to current drug-based control, given the increasing climate-driven changes in disease patterns and reports of treatment failure^11,18,45–47^. The fact that this strategy provides higher benefits in terms of risk reduction in Scotland and along the west coast of south England and Wales (Fig. 4a) is of interest as these areas: (i) are characterized by extensive grazing (Figure S1); (ii) are those where treatment is most common^44^ and drug resistance is prevalent^12^; and (iii) are either those associated with the highest liver fluke prevalence historically (e.g.^41^ and Fig. 1), or where the disease is expanding rapidly with warming climates^40^. Therefore, these are areas where treatment is particularly expected to become unsustainable in the future. The percentages of land to fence off, to achieve at least the same risk reduction as that obtained with treatment, are not unsubstantial, ranging from approximately 20% of catchment area up to more than 40% on the flattest regions, on average (and higher in wetter rather than drier years, Figure S2). This may make this intervention seem un-economical or impractical under current conditions. However, these values depend on the treatment option implemented, and our current scenario reflects the maximum reduction in risk obtainable with the most efficient drugs available, assuming no resistance (Fig. 3). In reality, farmers may treat at different times, with different (and often combinations of) products, that have lower efficacy and increasing resistance^43,44^. Moreover, the percentages we calculate represent the proportion of land to fence off to match or outperform treatment using this strategy *alone*, while -in the real world- temporary fencing would most likely be implemented in combination with (targeted) treatment as part of an integrated approach, still contributing to reduce reliance on drugs and delaying development of resistance^18^. On another note, our hydro-epidemiological mechanistic modelling approach could also be expanded to evaluate potential side benefits of management strategies beyond disease risk reduction, providing a holistic perspective that has so far been elusive (e.g. hydrologic ecosystem services could be derived from re-using fenced off high-risk areas for tree planting, which in turn might also reduce flood risk or create additional habitats for flora and fauna), and/or to inform novel fenceless grazing systems (by guiding the identification of high-risk areas to exclude from grazing through invisible GPS fences) enabling dynamic management of livestock and disease risk under highly variable conditions^49,50^. The second form of environmental disease management we investigate, which is drainage, is also often mentioned in the literature as a possible alternative to treatment, but it is also more controversial for multiple reasons. Our results suggest that a relatively small increase in drainage compared to current conditions (reducing the amount of water that contributes to soil saturation at the catchment scale) could actually help achieve risk reductions similar to those we can now obtain through drug administration across UK regions (Fig. 4b). While implementation of drainage in our model is at the catchment level, without differentiating between areas more and less prone to saturation, practical implementation of this strategy would require different proportions of land to be drained in flatter vs. higher relief catchments. A detailed assessment of the costs that such an intervention entails would also be needed to appropriately evaluate its economic feasibility. In the UK, current agri-environment programs, such as the Environmentally Sensitive Area scheme operated by the Department for Environment, Food and Rural Affairs, are increasingly discouraging drainage for environmental reasons, e.g. to preserve wetlands providing habitats to endangered flora and fauna^39^. Moreover, despite its known potential usefulness against liver fluke, this strategy is often considered prohibitively expensive^45–47^. On the other hand, some permanent artificial land drainage channels are already in place in England and Wales, including in low-lying areas in East Anglia, which are not accounted for in our analysis^51,52^. Also, drainage might become a competitive option for risk reduction in the long-run when farmers are increasingly faced with changing climatic conditions and drug resistance. For example, altered rainfall and temperature patterns might induce such frequent use of treatment in some areas, that drug administration becomes too expensive and ineffective, contributing to drainage emerging as a more valuable alternative. Ultimately, the optimal strategy will likely be farm-specific and depend not only on agricultural policy, but also on local herd, logistic and economic factors (that could be included in HELF for providing decision support at farm level) like the long-term costs/benefits of less intensive disease control strategies^18,44^.

In conclusion, our work demonstrates the feasibility and promotes the uptake of environmental management as a control strategy against fasciolosis in livestock in the UK, but has wider implications in the fight against environmentally transmitted infectious diseases anywhere^1^. It is increasingly acknowledged that future disease transmission cannot be eradicated with the use of drugs alone^9,37^. We show that mechanistic hydro-epidemiological models provide an integrated approach to investigating disease dynamics, which recognizes the complexities underpinning transmission, and which can quantify the role of disease risk drivers, including weather-landscape interactions^6,7^. Such deeper understanding of how meteorological and on-the-ground environmental processes shape disease epidemiology is key for informing new and sustainable solutions to infectious disease control in our changing world. We demonstrate that we can test what-if scenarios including different environmental interventions and management strategies over large space–time domains, which is crucial to potentially support their inclusion in national long-term plans for optimal disease control. These advancements address an urgent need as global climate change increasingly alters disease seasonality and spread^14–17^, and drug resistance develops rapidly^10,13^.

## Methods

### Data

The dataset we use includes meteorological, hydrological and Digital Elevation Model (DEM) data. Our domain consists of 935 hydrological catchments across Great Britain (i.e. England, Wales and Scotland) for which both meteorological and hydrological data are available for a recent decade, 2006–2015. Gridded (1 km resolution) daily time series of observed rainfall and min/max temperature are obtained from CEH-CHESS^53^. For each catchment, spatially averaged rainfall and min/max temperature time series are derived from this gridded dataset by considering grid cells that overlap with the catchment area. Streamflow data for the same 10-year period are obtained for all catchments from the National River Flow Archive^54^. Finally, gridded DEM data for Great Britain are obtained from NextMap, with spatial resolution of 50 m^55^, and used as a basis for digital terrain analysis to derive a topographic map for each catchment as in^56^ (see DEM data and catchments over Great Britain, together with a land cover map, in Figure S1).

### Disease risk model

We simulate disease risk using the recently-developed and tested Hydro-Epidemiological model for Liver Fluke (HELF)^24^. HELF mechanistically describes (at a daily time-step) how the impact of rainfall on the parasite life cycle is mediated by environmental characteristics through the process of soil moisture, known to directly drive disease transmission mainly due to its control on the habitat of the liver fluke’s intermediate snail host^5, 42^. The model, which assumes that topographic variability is the strongest landscape control on soil moisture and thus snail habitat distribution, estimates the propensity of an area to saturate through calculation of a Topographic Index ($TI$), and discretizes the distribution of $TI$ values of a catchment into classes, from the highest, most prone to saturation, to the lowest, assumed least likely to saturate^57^. The saturation state of each class then becomes an input to the parasite life-cycle model component of HELF, which uses it, together with air temperature, to calculate the abundance of metacercariae on pasture, i.e. the parasitic stage that, when ingested, infects grazing animals. This abundance is thus an indicator of environmental suitability for disease transmission to grazing livestock (also referred to as “disease risk” throughout the paper for the sake of simplicity). HELF is run over each of the 935 catchments in our domain for 2006–2015 using the dataset described above, while assuming a scenario of continuous livestock grazing and no disease management, as explained in^24^. Simulations for the first year are discarded as warm-up period, so that soil moisture states in the model can establish themselves. Disease risk results are then aggregated from daily to seasonal values (mean over July–September for summer and January–March for winter), as well as from $TI$ classes to the catchment scale (mean abundance of infective metacercariae, weighted based on the frequency of $TI$ classes). Details on the model set-up for application over Great Britain and on model calibration are given in SI.

### ANOVA

ANalysis Of VAriance (ANOVA) is a mathematical technique for partitioning the observed variance in a variable of interest (response variable) into contributions from individual drivers (factors) and their interactions. It has been widely used for different applications including for uncertainty estimation in climate change impact studies and for dominant control analysis (e.g.^58,59^). The response variable we focus on in our study is the seasonal catchment-average disease risk simulated using HELF (mean over the 9 years of simulation period, excluding 2006 for warm-up). The factors we use for variance decomposition are air temperature, potential evapotranspiration, rainfall, number of rainy days and topography (specifically, the mean of $TI$ values over each catchment), as well as their two-way interactions. In order to perform ANOVA, each factor is grouped into two levels, each with a similar number of catchments and level of disease risk variability (to satisfy the underlying assumption of homogeneous variances in ANOVA). This set up allows us to have multiple response variable observations for each combination of factor levels, making it possible to estimate the contribution of interactions. In ANOVA, the total variation in the response variable, to be attributed to the different factors, is expressed through the total sum-of-squares. This is split into main effects, corresponding to individual drivers, and interaction terms, related to non-additive or non-linear effects^58^. Therefore, the contribution of each factor to disease risk variability can be calculated as the proportion of its (partial) sum-of-squares and the total sum-of-squares (multiplied by 100 to get a percent contribution). The higher the contribution, the more the factor plays a key role in controlling disease risk. More details are provided in SI. We carry out the analysis at the regional scale to better capture the spatial distribution of dominant disease risk drivers. Specifically, we divide Great Britain into nine regions, as much as possible resembling the standard areas for which the UK NADIS currently provides forecasts of liver fluke risk based on a widely-used empirical climate-based model^48^. These, in turn, are based on the districts employed by the MetOffice when generating climatologies for the UK^60^: South East of England (SE), East Anglia (EAng), South West of England and West Wales (SW), the rest of Wales and the Midlands (Mid), North West and North East of England (NW and NE), and, finally, West, East and North of Scotland (WScot, EScot, NScot).

### Disease control strategies

Using HELF, we implement disease control strategies as follows:

1. Treatment. A recent survey throughout Great Britain and Ireland shows that almost 70% of farmers routinely treat their animals against liver fluke^44^. Triclabendazole is the most common drug used, which can reach above 90% efficacy against all parasitic stages in livestock, preventing contamination of pasture for up to 12 weeks. Based on this, as well as on available industry guidelines (e.g.^43^), we assume farmers treat animals twice per year, once on January 1^st^ and a second time on April 1^st^, using triclabendazole and under the hypothesis of no resistance. This approach is meant to reflect the maximum reduction in disease risk that can currently be achieved in the field using treatment (other available products reduce pasture contamination for shorter periods^43^ and the presence of resistance would reduce the efficacy of drugs in general). More details on this and on how the strategy is implemented within HELF are provided in SI.
2. Fencing off high-risk areas to prevent animals from grazing during high-risk periods. Traditionally, the period at highest risk of infection in the UK is in late summer, after temperatures have become generally more favorable for the parasite life cycle to progress within the snail hosts, yielding infective metacercariae on pasture^38^. The areas at highest risk are those most prone to saturation (i.e. those particularly flat, at the bottom of valleys), which are able to support high snail populations as well as other critical stages of the life cycle^5^. Therefore, we simulate this intervention by sequentially removing summer infective metacercariae from $TI$ classes (i.e. setting them to zero) starting from the $TI$ class with the highest value, which will saturate first. Then, for each catchment, by using the reduction in risk level achieved through treatment as a comparison, we estimate the percentage of area we would have to fence off if we wanted to obtain the same level or lower through fencing. Results are evaluated at the regional level using the 9 administrative areas defined above.
3. Drainage to permanently reduce soil moisture. For each catchment, we simulate the effect of drainage by varying a parameter within the hydrological model component of HELF that represents the catchment-level transmissivity of the soil when saturated to the surface (parameter LnTe in^24^). In the model, by increasing the value of this parameter, we increase the catchment contribution to discharge from the subsurface and decrease soil moisture in the catchment (here, in contrast to the implementation of fencing, we cannot assume that areas most prone to saturation are drained first, and the effect of drainage as implemented is at the catchment scale). The reduction in soil moisture, in turn, decreases habitat suitability for the snail hosts and free-living parasitic stages, and therefore reduces the abundance of infective metacercariae on pasture. Specifically, for each catchment in our domain, we increase this parameter by 1–50% of its calibrated value, using 20 uniformly distributed values, and run HELF with each of these, while leaving all other model parameters set to their calibrated values. Then, we study the percentage increase in drainage we would need to best match the risk level achieved through treatment and, again, evaluate results at the regional level.

## Supplementary Information

Supplementary Information.

## Data Availability

Underlying datasets are publicly available and referenced within the paper.
