# Supplementary material for: Discovering environmental management opportunities for infectious disease control
Source: Sci Rep. 2021 Mar 19;11:6442. doi: 10.1038/s41598-021-85250-1 (PMC7979760; doi:10.1038/s41598-021-85250-1)
Supplement: Supplementary file 1 — Supplementary Information. [file 41598_2021_85250_MOESM1_ESM.docx]

Supplementary Information (SI) for

**Discovering environmental management opportunities for infectious disease control**

Ludovica Beltrame^1*^, Hannah Rose Vineer^2^, Josephine G. Walker^3^, Eric R. Morgan^4^, Peter Vickerman^3^, and Thorsten Wagener^1,5,6^.

*^1^ Department of Civil Engineering, University of Bristol, Bristol, UK*

^2^ *Department of Infection Biology and Microbiomes, Institute of Infection, Veterinary and Ecological Sciences, University of Liverpool, Liverpool, UK*

^3^ *Bristol Medical School, University of Bristol, Bristol, UK*

^4^ *School of Biological Sciences, Queen’s University Belfast, Belfast, UK*

^5^ *Cabot Institute for the Environment, University of Bristol, Bristol, UK*

*^6^ Institute for Environmental Science and Geography, University of Potsdam, Potsdam, Germany*

^*^ [ludovica.beltrame@bristol.ac.uk](mailto:ludovica.beltrame@bristol.ac.uk)

**Supplementary text**

Additional information on fasciolosis

Fasciolosis is a widespread zoonotic disease caused by infection with two species of parasitic flatworms, or trematodes, of the genus *Fasciola*, which affect the liver (hence the common name liver fluke): *F. hepatica*, originally from temperate regions in Europe and America, and now expanded globally, and *F. gigantica*, which lives in tropical areas of Africa and Asia. Two hosts are required for these parasites to complete their life cycle. The primary host range is broad and includes several mammals (such as cattle, sheep, goats, equines, camelids and marsupials), including humans. Intermediate hosts are typically amphibious mud snails in the case of *F. hepatica*, and freshwater snails in the case of *F. gigantica*, both of the family Lymnaeidae. In the UK, fasciolosis mainly affects sheep and cattle (primary hosts), which get infected with *F. hepatica*, transmitted through *Galba truncatula* snails (intermediate hosts).

The disease has strong environmental components to its transmission, as a significant proportion of the parasite life cycle takes place in the environment, including the obligatory passage through the snails as well as free-living stages that can persist a long time outside of the hosts. Specifically, the life cycle of liver fluke unfolds as follows. Adult flukes in the bile ducts of infected animals produce eggs that are shed onto pasture through faeces. On pasture, eggs develop at temperature-dependent rates until they hatch into miracidia when both temperature and soil moisture conditions are suitable. Miracidia are short lived: they either find a snail host or die within 24 hours. European *G. truncatula* snails typically live along the edges of small ponds or ditches and on wet pastures, as poorly-drained areas subject to alternate flooding and desiccation provide their ideal habitat (1). They are also affected by temperature, and are known to hibernate with cold weather and aestivate during hot dry periods. Development of the parasitic stages within snails may be halted at these times, but, eventually, flukes multiply and, after about six to eight weeks, depending on temperature, large numbers of cercariae are released in the environment. These encyst on grass to form infective metacercariae, which survive and retain infectivity based on temperature, with moderate weather being most favorable. When grazing animals ingest metacercariae, the immature flukes migrate into their liver, reach the bile ducts, and the life cycle resumes.

Regarding liver fluke models, most current disease risk forecasting tools only assume meteorological drivers of fasciolosis, neglecting how their effects on disease transmission may be modified by on-the-ground environmental characteristics. For example, the Ollerenshaw Index, developed in the 1950s but still widely used to warn farmers about potential high-risk years across Europe, is based on a relationship between weather characteristics and incidence of acute fasciolosis data found on the island of Anglesey, Wales (UK), over the period 1948-1957, and is commonly calculated as follows: $M_{t}=n \left( \frac{R}{25.4}-\frac{P}{25.4}+5 \right)$. Where:

- $M_{t}$ is the monthly index of risk of infection with fasciolosis;
- $n$ is the monthly number of rainy days (above 1mm);
- $R$ is the monthly rainfall [mm];
- $P$ is the monthly potential evapotranspiration [mm].

However, it is environmental factors that, as opposed to weather, may be modified towards risk reduction. On the other hand, existing studies that do consider environmental drivers of fasciolosis and management factors, do so empirically, e.g. correlation-based models or investigations of the impact of environmental interventions in the field at specific times and locations (e.g. 1-4). However, empirical approaches are unsuitable for capturing climatic-environmental impacts beyond historically-observed variability and cannot be used to test control strategies through what-if analyses. The fact that most existing disease risk models neglect the role of on-the-ground environmental drivers or only consider them empirically is not only the case for fasciolosis, but is also common for other environmentally-transmitted diseases (e.g. see (5), and references therein). For fasciolosis, the recent mechanistic Hydro-Epidemiological model for Liver Fluke (HELF), which explicitly represents how weather impacts are mediated by topography and soil moisture, was developed specifically to assess risk of infection under changing conditions and to evaluate disease control scenarios including environmental interventions (e.g., what is the extent of areas prone to transmission (i.e. of pasture contaminated with infective metacercariae) that we need to fence off to limit livestock exposure and therefore reduce disease risk? And, thus, how effective is acting on grazing management through fencing as a disease control strategy?).

Regarding disease control, drug treatment in the context of fasciolosis refers to anthelmintic treatment (i.e. drugs against helminths –or parasitic worms like liver fluke). Despite several trials in recent years, no commercial vaccine is yet available for prevention of fasciolosis. Therefore, current control is largely based on the use of treatment (mainly triclabendazole, for both people and animals). However, disease control has proven to be intractable in areas where environmental conditions favor transmission and is increasingly compounded by drug resistance. Farmers are already encouraged to use alternative products to triclabendazole (that reduce egg production for a shorter period of time), as a large proportion of farms have now observed treatment failure. Going forwards, it is expected that, as climate change increases opportunities of transmission and resistance undermines control based solely on treatment, complementary strategies (such as targeted drug administration combined with environmental management) will become paramount towards reducing disease burdens.

Additional information on the application of HELF across Great Britain

Setting up HELF to run over Great Britain involves introducing a loss term in the hydrological component of the model presented by Beltrame et al. (2018), to better represent the hydrology in case of groundwater-dominated catchments. Specifically, to account for groundwater that may not reach the river in catchments with low runoff ratio (i.e. with low ratio of runoff to rainfall), we introduce an extra parameter in the previous model and assume that, at each time step, storage in the saturated zone (i.e. the groundwater) not only is refilled by vertical flow from the root zone and drained by subsurface flow, but also decreases linearly with storage through this extra parameter (e.g. see (6)).

With regard to model calibration, on one hand, we estimate parameters for the hydrological component of HELF by using signatures derived from streamflow observations (namely, runoff ratio and central slope of the flow duration curve, calculated as in (7-8)). On the other hand, no additional calibration of the epidemiological component of the model is performed compared to Beltrame et al. (2018), since nation-wide continuous disease prevalence data are not available. Instead, we use one (mean) parameter set from the ranges obtained after application of the expert-driven rules described in Beltrame et al. (2018), assuming liver fluke life-history parameters to be relatively constant across the UK. Similar assumptions have been made in previous studies focused on regions of similar ecology and single snail host species (e.g. 9), which are conditions consistent with our case.

Additional information on ANOVA

To investigate the relative contribution of environmental drivers and their (two-way) interactions to disease risk variability across Great Britain, we perform a 5-way ANOVA experiment for each of our 9 regions (assessed at the 95% confidence level). This means that, for every region, we consider 5 potential sources of variation (factors), including their two-way interactions, and one response variable. The 5 factors we use are: number of rainy days (RD), rainfall (R), temperature (T), potential evapotranspiration (P) and topography (TOPO). The response variable we are interested in is disease risk, as modelled using HELF. Regarding ANOVA underlying assumptions, in our dataset, the correlation between the considered factors is below 0.7 (i.e. |correlation coefficient|≤0.7), which has been shown to be appropriate for avoiding significant factor dependency-related problems in the literature (10), while satisfaction of homogeneous variance and population normality are checked by verifying boxplots. In order to perform ANOVA, each factor needs to be classified into a number of levels. In our case, each of the 5 factors has 2 levels (low and high), which means we have a 2^5^ experiment, i.e. 32 combinations of factor levels, each associated to an observation of the response variable. The fact that all these combinations are present in our dataset makes our experiment “fully-crossed”, which allows us to study the effect of interactions between factors on disease risk. However, the number of observations we have for each combination may differ, which often makes our ANOVA test “unbalanced”. As, when applying ANOVA to unbalanced data, the resulting sum-of- squares will depend on the order in which the sources of variation are considered, instead of performing one test per region only, we perform one test for each possible order of our 5 factors, and then evaluate the resulting ranking of drivers on average across these. Table S1 summarizes regional ANOVA results obtained with one specific order of drivers, as an example. It includes sum-of-squares and p-values associated with individual drivers and two-way interactions, as well as relative proportions of the variance that remain unaccounted for (i.e. the error terms). Results are statistically significant (i.e. factors are important drivers of disease risk) if their p-value is ≤ 0.05 (highlighted in green). We find that topography (TOPO) results significant in all three Scottish regions (NScot, WScot and EScot), and that significant interaction terms are not always necessarily interactions between weather factors only. For example, while in flat areas in the south east of the country (South-East of England (SE) and East Anglia (EAng)), it is the interaction between meteorological drivers themselves that explains most of the variability in disease risk (e.g. see RD x T for SE, and RD x T or R x P for EAng), along the west coast of England and Wales disease risk shows higher sensitivity to interactions between weather characteristics and topography (e.g. see P x TOPO for South West of England and West Wales (SW) and RD x TOPO for North West of England (NW)). Finally, we remark that, in this example, percent contributions do not always sum up to 100 and error terms are large. The former is due to the fact that partitioned variances do not always sum up to 1 when ANOVA tests are unbalanced. The latter may partly be affected by this, but also potentially confirms that the complex dynamics of disease risk cannot be explained by simple linear models of the explanatory variables.

Table S1. Regional 5-way ANOVA of Rainy Days (RD), Rainfall (R), Temperature (T), Potential evapotranspiration (P) and Topography (TOPO), and their two-way interactions, on disease risk modelled using HELF. Significant p-values (≤ 0.05) are highlighted in green.


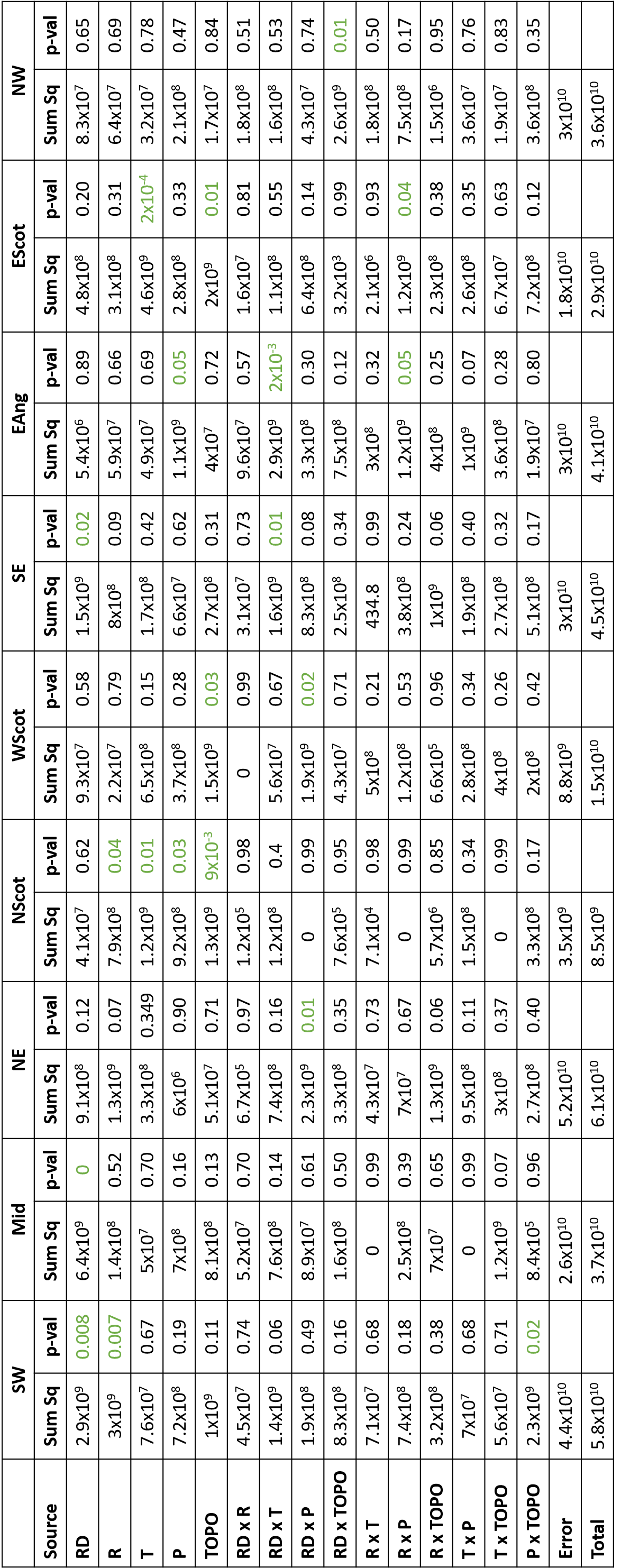


Additional information on the implementation of treatment

Our treatment scenario aims to reflect the maximum reduction in disease risk that can currently be achieved in the field using drug administration assuming no resistance. To this end, following Morgan et al. (2012) and available industry guidelines, we assume that farmers treat their sheep twice per year in winter/spring using triclabendazole. We focus on (adult) sheep as the most relevant animal group for defining our scenario because treatment of cattle usually aims more at protection rather than reduction of pasture contamination, and is more strongly constrained by management factors (11). Similarly, we assume treatment is administered in winter/spring because, although farmers may also treat in autumn, autumn treatments against immature fluke mainly aim to protect sheep from acute disease and serve to delay but not necessarily to decrease egg production. Finally, we assume the product of choice is triclabendazole because currently this is the most common drug used, which has 90% efficacy against all parasitic stages in livestock (i.e. mature and immature flukes), preventing contamination of pasture for 12 weeks, until newly ingested infective metacercariae mature and begin egg laying again (other available products have lower efficacy and reduce egg shedding for shorter periods). The strategy is implemented in HELF by modifying the egg scenario in input to the model. Specifically, instead of considering a scenario of continuous livestock grazing and no disease management (with 100 embryonic eggs introduced on pasture daily all-year-round (for every year and catchment)), we set egg counts to zero for 12 weeks starting 1st January and 1st April (i.e. over January-May) and allow eggs to linearly increase back to the constant value of 100 eggs/day over June.

**Supplementary figures**

Figure S1. Datasets for Great Britain (maps created using ArcGIS 10.7.1): Land Cover Map (LCM; from (12)), Digital Elevation Model (DEM) data, and hydrological catchments (including gauged and ungauged ones). The west coast is where most pasture grazing takes place in Great Britain, as well as where topographic variations are largest (following Scotland), whereas the south east of the country is mostly characterized by flat low-lying areas.


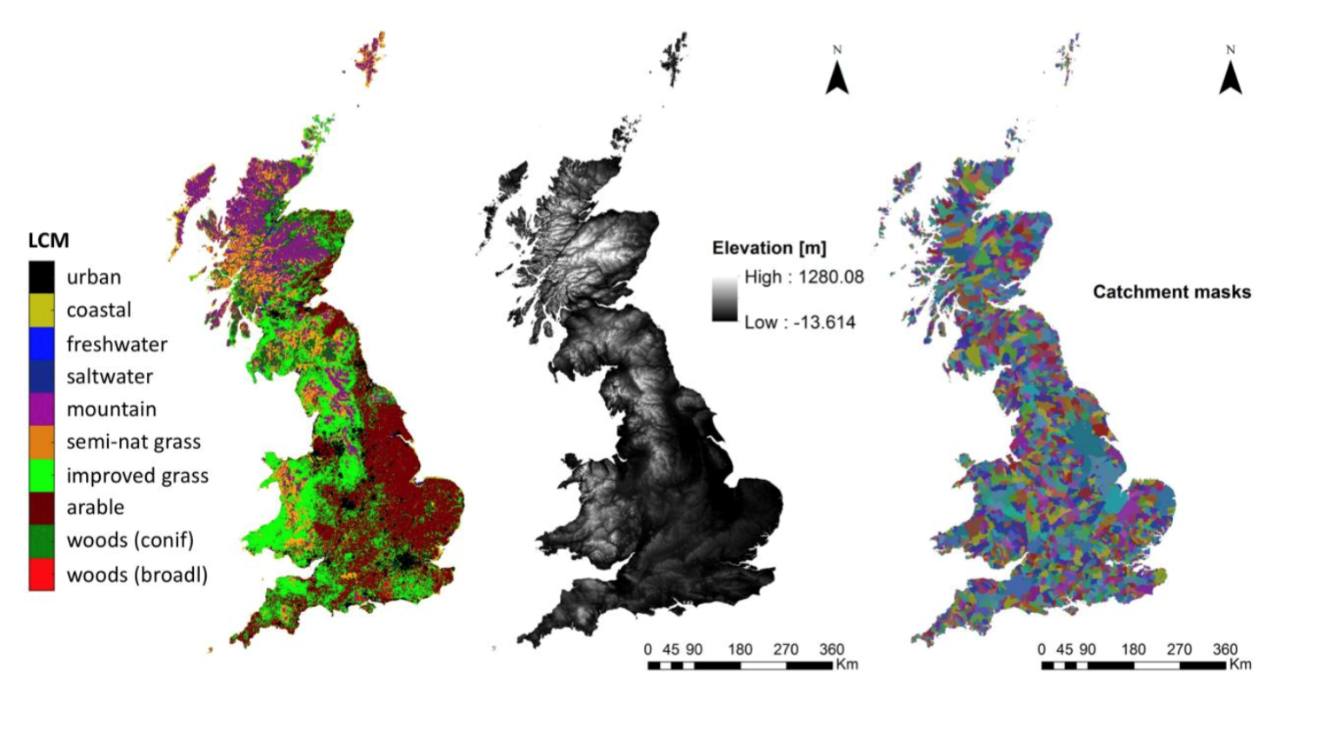


Figure S2. Percentage of catchment area that would need fencing off to reduce summer risk of infection by at least the same percentage achieved using treatment (on average across catchments within each region). Comparison of 2013 and 2014, which represent a relatively dry and a relatively wet year within our simulation period, respectively. Temporary fencing of high-risk areas appears to be more convenient in regions where topographic variations are larger (which is where saturated areas providing snail habitats will be more localized, e.g. along the west coast of south England and Wales and in the north of the country), rather than where the landscape is mostly flat (e.g. on low-lying areas in East Anglia), and, particularly, in relatively dry years (in North and East Scotland, where summer temperatures decrease from 2013 to 2014, percentages of land to fence off are lower in 2014. In fact, a colder summer will result in less favorable conditions for development of the parasite life cycle. In all other regions, the portions of land to fence off – to be at least as effective as treatment– are higher in wetter 2014, by 5.7% on average). These results are in line with recommendations of recent guidelines for liver fluke control. (Maps created using Matlab R2019a).


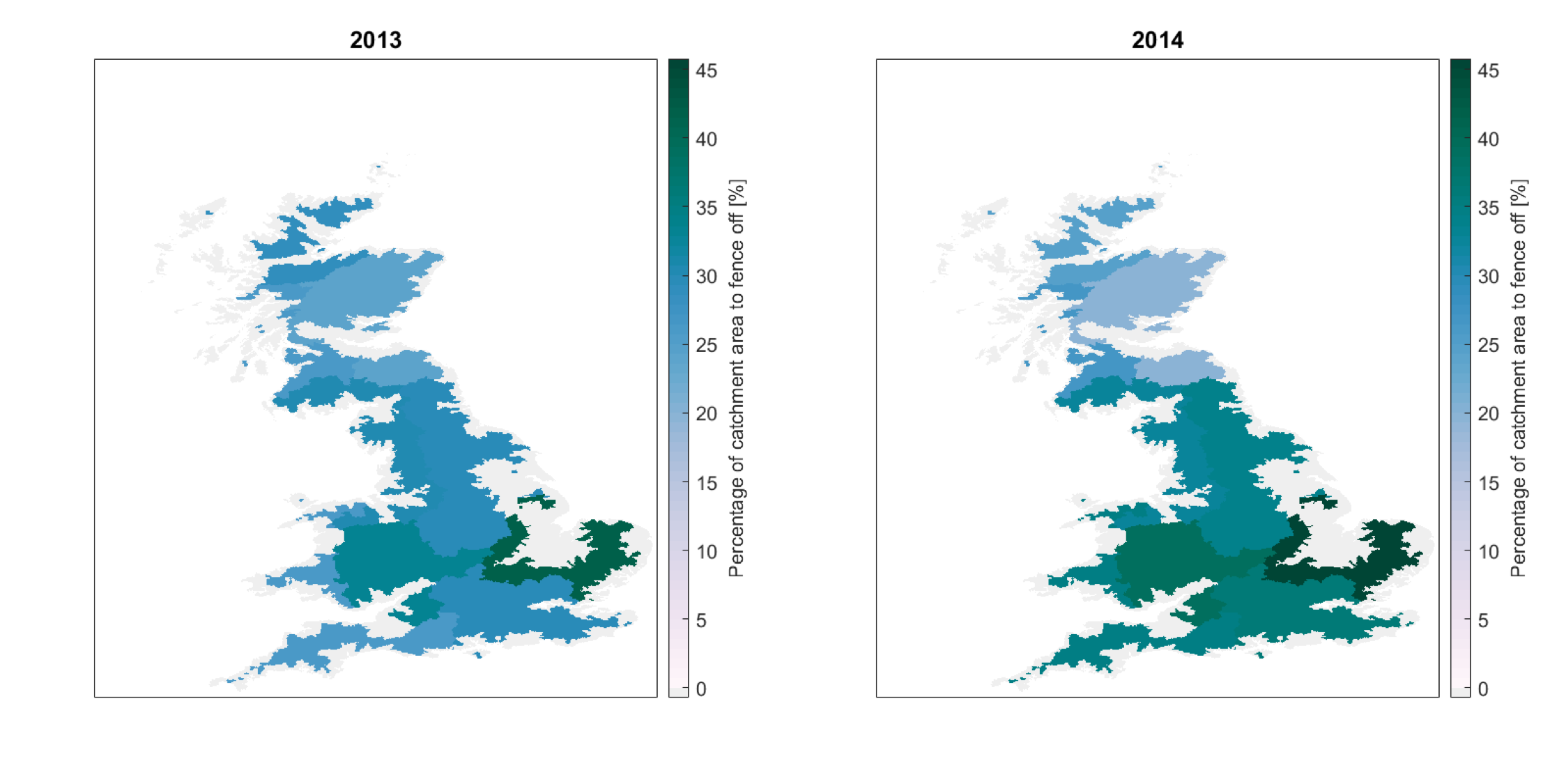


Figure S3. Percentage reduction in summer risk of infection achieved by fencing off portions of catchment area, starting from those most prone to saturation until the whole catchment is virtually fenced off, compared to the case of no intervention. For all 9 regions (defined in Figure 1 of the paper), percentages of catchment area on the x–axes are ordered by Topographic Index class, from the most to the least prone to saturation (i.e. from high to low Topographic Index value). Each line is the mean across catchments within the region for a certain year within the simulation period. Years are color-coded from that with the driest summer, in red, to that with the wettest summer, in blue. On one hand, the impact of fencing off the highest Topographic Index classes, which represent on average 5-10% of the catchment area, is similar across regions. These are presumably areas of a catchment that are saturated for much of the year (e.g. 13-14). On the other hand, the risk reductions achieved by fencing off larger portions of catchment area differ between regions (e.g. to reduce risk by 65%, as we currently obtain with treatment on average across the country, grazing should be avoided on large fractions of catchment area in East Anglia (~56%), but on smaller percentages in Wales/Midlands (e.g. ~38% for Mid), and even smaller percentages in Scotland (e.g. ~18% for NScot)). Similarly, risk reductions achieved through fencing can vary from year to year, presumably based on the dynamics of multiple weather controls (the one highlighted here, summer rainfall, seems to matter in some regions more than others but not consistently between years, suggesting that there are other factors playing a role, such as rainfall and temperature distributions over preceding months, rather than just during the season considered). Overall, by demonstrating how the effectiveness of this environmental intervention may differ with place (e.g. from hill to lowland areas) and year (e.g. with different weather conditions over summer and preceding months), these results also provide us with guidelines to help design targeted field studies for the future.


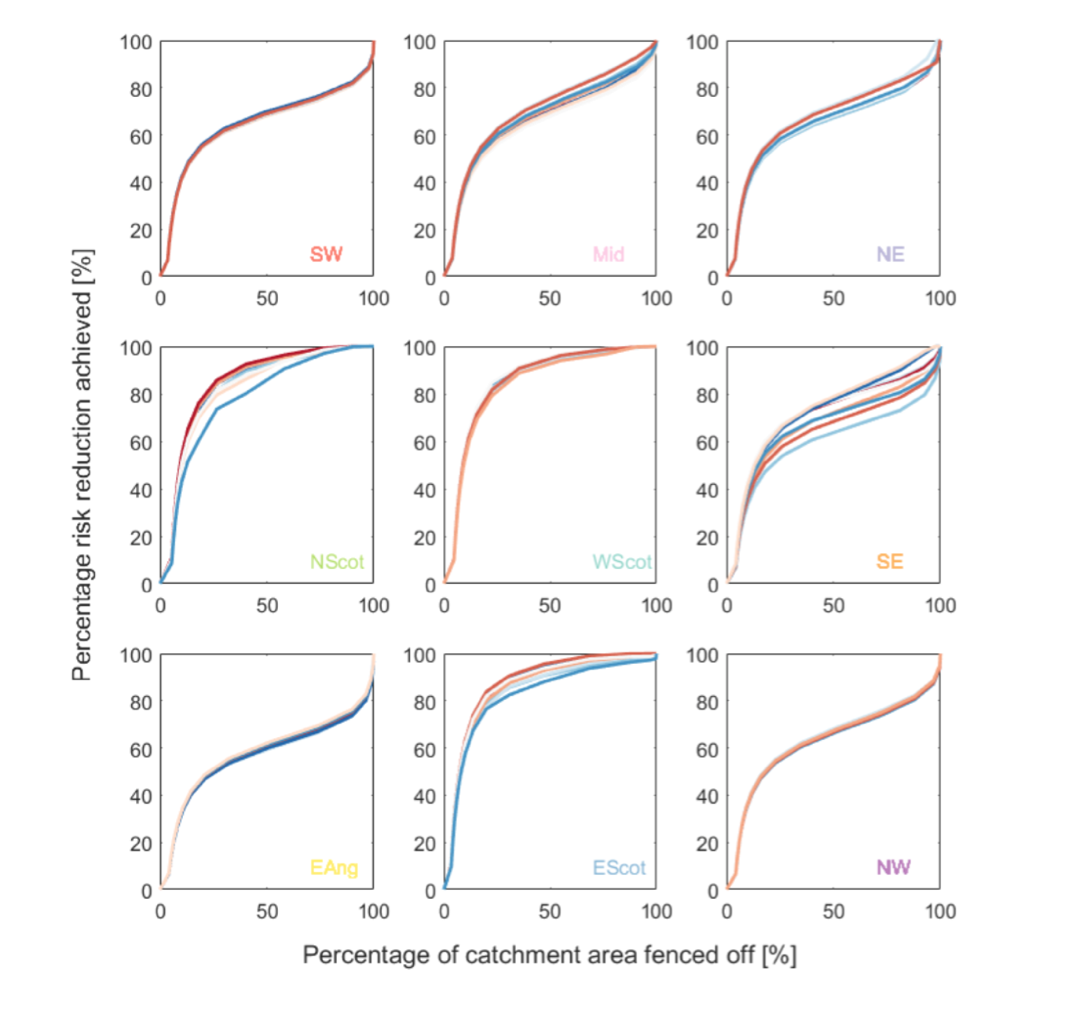


**SI References**

1. Charlier, J., Bennema, S. C., Caron, Y., Counotte, M., Ducheyne, E., Hendrickx, G., & Vercruysse, J. (2011). Towards assessing fine-scale indicators for the spatial transmission risk of Fasciola hepatica in cattle. Geospatial Health, 5(2), 239–245. http://doi.org/10.4081/gh.2011.176
2. Yilma, J. M., & Malone, J. B. (1998). A geographic information system forecast model for strategic control of fasciolosis in Ethiopia. Veterinary Parasitology, 78(2), 103–127. http://doi.org/10.1016/S0304-4017(98)00136-8
3. Bennema, S. C., Ducheyne, E., Vercruysse, J., Claerebout, E., Hendrickx, G., & Charlier, J. (2011). Relative importance of management, meteorological and environmental factors in the spatial distribution of Fasciola hepatica in dairy cattle in a temperate climate zone. International Journal for Parasitology, 41(2), 225–233. http://doi.org/10.1016/j.ijpara.2010.09.003
4. Howell, A., Baylis, M., Smith, R., Pinchbeck, G., & Williams, D. (2015). Epidemiology and impact of Fasciola hepatica exposure in high-yielding dairy herds. Preventive Veterinary Medicine, 121(1– 2), 41–48. http://doi.org/10.1016/j.prevetmed.2015.05.013
5. Rinaldo, A., Bertuzzo, E., Blokesch, M., Mari, L., & Gatto, M. (2017). Modeling Key Drivers of Cholera Transmission Dynamics Provides New Perspectives for Parasitology. Trends in Parasitology, 33(8), 587–599. http://doi.org/10.1016/j.pt.2017.04.002
6. Wagener, T., Wheater, H. S. & Gupta, H. V. (2004). Rainfall-Runoff Modelling in Gauged and Ungauged Catchments. https://doi.org/10.1142/p335
7. Sawicz, K., Wagener, T., Sivapalan, M., Troch, P. A., & Carrillo, G. (2011). Catchment classification: Empirical analysis of hydrologic similarity based on catchment function in the eastern USA. Hydrology and Earth System Sciences, 15, 2895–2911. http://doi.org/10.5194/hess-15-2895-2011
8. Yadav, M., Wagener, T., & Gupta, H. (2007). Regionalization of constraints on expected watershed response behavior for improved predictions in ungauged basins. Advances in Water Resources, 30(8), 1756–1774. http://doi.org/10.1016/j.advwatres.2007.01.005
9. Liang, S., Maszle, D., & Spear, R. C. (2002). A quantitative framework for a multi-group model of Schistosomiasis japonicum transmission dynamics and control in Sichuan, China. Acta Tropica, 82(2), 263–277. http://doi.org/10.1016/S0001-706X(02)00018-9
10. Dormann, C.F., Elith, J., Bacher, S., Buchmann, C., Carl, G., Carre, G., … Lautenbach, S. (2013). Collinearity: a review of methods to deal with it and a simulation study evaluating their performance. Ecography, 36(1), 27–46. https://doi.org/10.1111/j.1600-0587.2012.07348.x
11. Charlier, J., Soenen, K., De Roeck, E., Hantson, W., Ducheyne, E., Van Coillie, F., … Vercruysse, J. (2014). Longitudinal study on the temporal and micro-spatial distribution of Galba truncatula in four farms in Belgium as a base for small-scale risk mapping of Fasciola hepatica. Parasites & Vectors, 7(528). http://doi.org/10.1186/s13071-014-0528-0
12. Rowland, C. S., Morton, R. D., Carrasco, L., McShane, G., O’Neil, A. W. & Wood, C. M. (2017) Land Cover Map 2015 (vector, GB). NERC Environmental Information Data Centre. https://doi.org/10.5285/6c6c9203-7333-4d96-88ab-78925e7a4e73
13. Güntner, A., Uhlenbrook, S., Seibert, J., & Leibundgut, C. (1999). Multi-criterial validation of TOPMODEL in a mountainous catchment. Hydrological Processes, 13(11), 1603–1620. [http://doi.org/10.1002/(SICI)1099-1085(19990815)13:11<1603::AID-HYP830>3.0.CO;2-K](http://doi.org/10.1002/(SICI)1099-1085(19990815)13:11%3C1603::AID-HYP830%3E3.0.CO;2-K)
14. Güntner, A., Seibert, J., & Uhlenbrook, S. (2004). Modeling spatial patterns of saturated areas: An evaluation of different terrain indices. Water Resources Research, 40(5), W05114. http://doi.org/10.1029/2003WR002864
